# Unwrapping Neurotrophic Cytokines and Histone Modification

**DOI:** 10.1007/s10571-016-0330-y

**Published:** 2016-03-02

**Authors:** Cieron Roe

**Affiliations:** Brighton and Sussex Medical School, The Audrey Emerton Building, Eastern Road, Kemp Town, Brighton, BN2 5BE UK

**Keywords:** Neurotrophic cytokines, Histone modification, Myelination, Epigenetics, Therapy, Neurodegeneration, Oligodendrocytes

## Abstract

The conventional view that neuroinflammatory lesions contain strictly pro- and anti-inflammatory cytokines is being challenged. Some proinflammatory products e.g. TNF-α are crucial intermediates in axon regeneration, oligodendroglial renewal and remyelination. A more functional system of nomenclature classifies cytokines by their neuro ‘protective’ or ‘suppressive’ properties. Beyond the balance of these ‘environmental’ or ‘extrinsic’ signals, specific ‘intrinsic’ determinants of cytokine signalling appear to influence the outcome of axoglial regeneration. In this commentary, we examine the potential importance of cytokine-induced histone modification on oligodendrocyte differentiation. Neuroinflammation mediates the release of astrocytic leukaemia inhibitory factor (LIF) and erythropoietin (EPO) which potentiates oligodendrocyte differentiation and myelin production. Meanwhile, histone deacetylation strongly suppresses important inhibitors of oligodendrocyte differentiation. Given that LIF and EPO induce histone deacetylases in other systems, future studies should examine whether this mechanism significantly influences the outcome of cytokine-induced remyelination, and whether epigenetic drug targets could potentiate the effects of exogenous cytokine therapy.

## Background

Oligodendrocyte proliferation and differentiation is regulated by an intricate network of extrinsic signals and intrinsic genetic and epigenetic control mechanisms. These are in turn determined by the dynamic milieu of the central nervous system (CNS). Amongst an array of important extracellular signals, astrocytes release leukaemia inhibitory factor (LIF) and erythropoietin (EPO) which are neuroprotective cytokines that promote the differentiation of OLs and preserve the integrity of the myelin sheath (Ishibashi et al. 2006, 2009; Cervellini et al. 2013). In the ‘steady state’, neuronal depolarisation releases adenosine which stimulates the production of these cytokines (Ishibashi et al. 2006). This process amplifies during episodes of ‘axoglial stress’, e.g. following excitotoxicity or when TNF-α is produced by inflammatory infiltrates (Fischer et al. 2014; Moidunny et al. 2012). Previously, we discussed potential *genetic* elements that may influence the efficacy of exogenous cytokine therapy (Roe 2015). Surprisingly, the literature paid little attention to the interactions between cytokine signalling and mediators of *epigenetic* modification.

Epigenetic modifications are defined as “heritable alterations to the DNA architecture that do not change the nitrogenous base sequence”. The ‘epigenome’ describes a landscape of chemical modifications affecting the histone proteins that wrap DNA (histone acetylation and methylation), or more directly, the DNA itself (DNA methylation). Previous studies have shown that the process of (re)myelination is dependent on a variety of epigenetic remodelling mechanisms including histone modification, DNA methylation and miRNAs (Shen et al. 2005, 2008; Liu and Casaccia 2010). In this commentary, we explore the role of histone modification on oligodendrocyte differentiation, the potential for neurotrophic cytokines to induce these modifications and the scope for novel therapeutic targets to bolster remyelination in the context of neuroinflammatory disease.

## Commentary

**Fig. 1:**
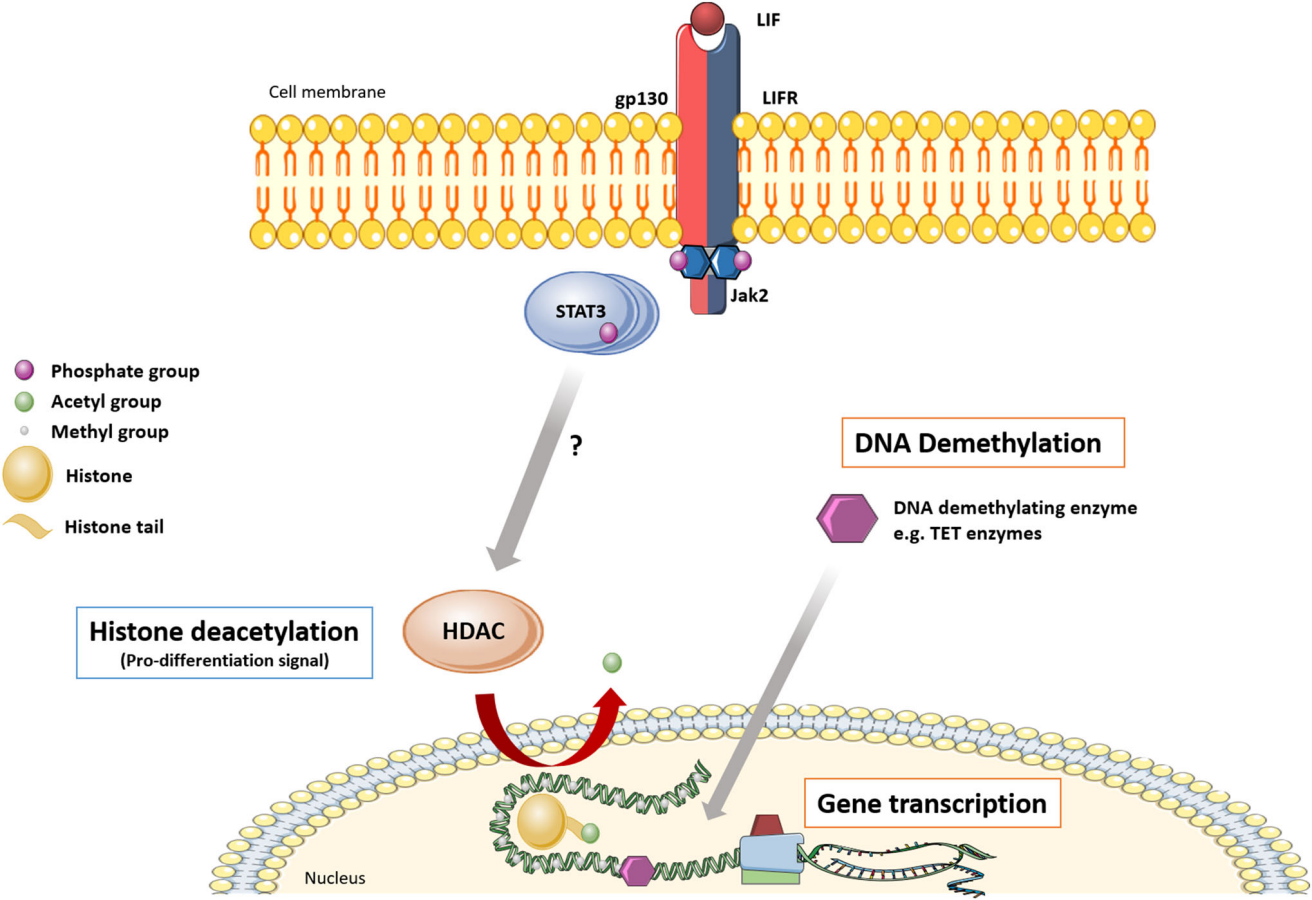
Proposed schematic of LIF-induced epigenetic ‘de-repression’ of OL differentiation. As in stem cells, LIF may induce HDACs that deacetylate histones associated with gene loci that inhibit oligodendrocyte differentiation. Deacetylation leads to chromatin compaction which prevents DNA demethylation and thus the transcription of genes that inhibit myelination

The conformational structure of chromatin determines the accessibility of genes. Acetylation of histone ‘tails’ reduces the positive charge that compresses the chromatin structure (Fig. 1). These modifications are determined by two key enzyme families; histone acetyltransferases (HATs) which acetylate histones and HDACs which remove the acetyl groups. The latter increases the electrostatic interaction to compress chromatin and hide regulatory gene elements. HDACs and HATs also (de)acetylate and physically interact with other nuclear proteins to form large transcriptional complexes that directly regulate gene expression.

In primary mouse oligodendrocyte progenitor cells, the capacity for myelination correlates directly with the activity of HDACs (Shen et al. 2008). Ye et al. found that HDAC1/HDAC2^−/−^ mice developed severe tremors and died at postnatal day 14 (Ye et al. 2009). Oligodendrocyte progenitor-specific markers PDGFRA and OLIG2 were absent at embryonic day 15.5, and markers of differentiated oligodendrocytes were absent from postnatal day 4 (Ye et al. 2009). In addition, primary cortical oligodendrocyte precursors underwent differentiation arrest when cultured in vitro (Ye et al. 2009). As Li et al. show, HDAC1 and HDAC2 are critical for optimal oligodendrocyte specification and differentiation (Li and Richardson 2009). These HDACs were associated with transcriptional control regions believed to inhibit oligodendrocyte myelination. Furthermore, the expression of these inhibitory genes decreased in line with HDAC activity (Shen et al. 2008). Interestingly, chromatin is predominantly acetylated in primary oligodendrocyte progenitors whereas it grossly deacetylates during differentiation (Shen et al. 2005; Liu et al. 2007; Marin-Husstege et al. 2002). Indeed treatment with HDAC inhibitors arrests maturation (Shen et al. 2005; Liu et al. 2007; Marin-Husstege et al. 2002). These studies support Emery’s ‘de-repression’ theory which argues that environmental cues e.g. inflammation prompts the attenuation of inhibitors of differentiation, which in this context is open chromatin and demethylated DNA (Fig. 1) (Emery 2010).

LIF is well known for its function in maintaining the pluripotency of embryonic stem cells. This occurs through the Janus kinase/signal transducer and activator of transcription 3 (Jak/Stat3) pathway. McCool et al. showed that withdrawal of LIF leads to a global increase in histone acetylation that mimicked the effect of trichostatin A, a histone deacetylase inhibitor (McCool et al. 2007). These data could suggest that LIF-induced STAT3 regulates the action of epigenetic modifiers which control access to important genes (Fig. 1). These effects fit with the pattern of HDAC levels required to mediate (re)myelination and this may in part, explain LIF’s promyelinating effects. Yamamura et al. showed that FK228 (a HDAC inhibitor) suppresses the anti-apoptotic effects of EPO on erythroid precursor cells (Yamamura et al. 2006). The authors speculated that HDAC inhibition may block an EPO signalling pathway, indicating that EPO induces HDACs. Again, given EPO’s capacity to promote oligodendrocyte differentiation, we postulate that, as in erythrocytes, EPO may induce HDACs to attenuate inhibitors of myelin gene expression. In addition, Ye et al. showed that HDAC1 and HDAC2 stimulate oligodendrocyte differentiation by directly antagonizing the inhibitory action of Wnt signalling (Ye et al. 2009).

## Conclusion

Epigenetic processes clearly influence the outcome of (re)myelination. Histone deacetylation is associated with the repression of important inhibitors of oligodendrocyte differentiation and neurotrophic cytokines such as LIF and EPO have demonstrated a potential to induce these HDACs in other systems. Future research should specifically examine the epigenetic processes that occur downstream of LIF and EPO in oligodendrocytes. This could lead to a novel therapeutic approach to demyelinating disease that incorporates epigenetic drug targets e.g. HDACs to bolster the efficacy of exogenous cytokine therapy.

## Abbreviations

TNF-α: Tumour necrosis factor-alpha
LIF: Leukaemia inhibitory factor
EPO: Erythropoietin
HDACs: Histone deacetylases
CNS: Central nervous system
HATs: Histone acetyltransferases
PDGFRA: Platelet derived growth factor receptor alpha
OLIG2: Oligodendrocyte transcription factor 2
Jak: Janus kinase
STAT3: Signal transducer and activator of transcription 3
